# Acoustic Analysis of Soundproofing Materials Using Recycled Rubber from Automobiles

**DOI:** 10.3390/ma18133144

**Published:** 2025-07-02

**Authors:** Miroslav Badida, Miriam Andrejiova, Miriama Pinosova, Marek Moravec

**Affiliations:** 1 Department of Business Management and Economics, Technical University of Košice, 042 00 Košice, Slovakia; miroslav.badida@tuke.sk; 2 Department of Applied Mathematics and Informatics, Technical University of Košice, 042 00 Košice, Slovakia; miriam.andrejiova@tuke.sk; 3 Department of Quality, Safety, and Environment, Technical University of Košice, 042 00 Košice, Slovakia; miriama.pinosova@tuke.sk

**Keywords:** recycled rubber crumb, acoustic properties, transmission loss, measurement, impedance tube

## Abstract

This article provides a comprehensive analysis of the acoustic properties of recycled rubber crumb, examined in two forms—loose granular and compacted specimens. The aim was to compare their acoustic properties depending on the size of the fraction, the thickness of the sample, and the degree of compaction, with measurements performed using a model BSWA SW433 impedance tube in the frequency band 100–2500 Hz. Experimental samples of recycled rubber crumb were prepared with various thicknesses (2, 4.5, and 7 cm) and of various fractions (0–4 mm), and the granular samples were compacted under a pressure of 250–750 kPa. The results showed that the highest transmission loss (TL) is achieved by fine fractions at higher pressure and with greater sample thickness; Fraction 1 (below 1 mm) at a pressure of 750 kPa and a thickness of 7 cm had the best acoustic properties. Through regression analysis, mathematical models of the dependence of transmission loss on the monitored parameters for all types of samples (granular/compacted) were created. The regression analysis confirmed that the thickness, pressure, and size of the fraction significantly affect the acoustic properties of the material. Recycled rubber crumb therefore represents an efficient and environmentally sustainable alternative to traditional insulation materials, and optimizing its parameters enables a wide range of practical acoustic applications in construction, transport infrastructure, and manufacturing industries.

## 1. Introduction

The acoustic properties of materials play a critical role in developing and implementing noise control measures across various industries. Analyzing these properties is essential for effective noise mitigation [1,2]. Optimizing the acoustic environment depends on selecting suitable construction and sound-absorbing materials, as well as the methods used for their application. Environmental sustainability is also a key consideration in designing acoustic materials, especially given the increasing consumption of raw materials, the growth of plastic waste in ecosystems, and global environmental pollution. Recycling rubber waste is one of the main challenges in end-of-life vehicle recycling. Due to their unique physical and chemical properties, recycled rubber materials can provide significant benefits in acoustic and thermal insulation, as well as vibration control, when used appropriately.

Desarnaulds et al. [3] described traditional insulation materials, which are primarily used in building acoustics because of their favorable environmental and acoustic properties. These materials fall into two main categories: natural materials, such as cotton, cellulose, or hemp, and recycled materials, including rubber, cork, glass, textiles, and other secondary raw materials. Natural materials have a low environmental footprint throughout their life cycle. Their main advantages are complete recyclability and biodegradability. Additionally, most of these materials do not require binders, as they can be compacted using a pressing device [4,5,6]. McGinnes et al. [7] highlighted the benefits of natural materials, such as straw, in the construction industry, emphasizing their availability, ease of use, and environmental friendliness. They found that the sound absorption of these materials is comparable to that of mineral wool or glass fibers. However, D’Alessandro et al. (2017) refuted this claim, showing that straw bale walls are weak sound insulators at low frequencies and do not meet building code requirements [8]. The authors [4] pointed out that natural and recycled materials offer cost and environmental advantages over synthetic materials. However, the acoustic properties of many natural materials remain insufficiently studied.

A new approach to the reuse of shredded recycled rubber was presented by the authors in a study [9], in which they highlighted the growing problem of the large number of worn-out tires generated each year. As part of their research, they developed a new material with potential applications in construction as both an acoustic and thermal insulation element. They also emphasized that the use of crushed recycled rubber in these areas is only sporadically addressed in the available literature, with the exception of a few publications, such as the studies by Sambucci et al. [10,11,12].

Akiwate et al. [13] investigated the acoustic properties of porous materials produced using 3D printing technology. Labašová and Ďuriša [14] evaluated the acoustic absorption coefficients of various single- and double-layer materials, such as cork, felt, and polyethylene. Sandu et al. [15] tested 17 different materials—including recycled options such as cinder, polystyrene, cork, balsa wood, and bird feathers—to identify those with the highest potential for noise reduction. The authors [16] investigated the sound insulation properties of recycled textile fiber composites, evaluating the acoustic properties using an impedance tube in the frequency range of 100–3200 Hz. Their results showed that absorption improved with increasing composite layer thickness. Research by the authors [17] on the acoustic properties of concrete, mortar, and stabilized soil revealed that these materials achieved the highest absorption at low frequencies (100–500 Hz). Concrete exhibited the highest sound absorption coefficient (0.94), followed by mortar (0.85) and stabilized earth (0.74). These findings highlight significant differences in the acoustic properties of individual materials, which are important considerations for their application in the construction industry, particularly regarding sound insulation.

The impedance tube has been used in experimental studies by Suhanek M. et al., Pierre J. et al., Koruk H., Tan W.H. et al., and Neto B.N. et al. [18,19,20,21,22]. These studies demonstrate the versatility of impedance tube measurements for evaluating the acoustic properties of various recycled materials, highlighting their potential applications in sustainable building and noise reduction solutions.

The selection of recycled rubber granulate from automotive tires as the primary material for this study was motivated by environmental sustainability, as tire recycling represents a significant challenge in automotive waste management. The material is also readily available due to a well-developed recycling infrastructure and is cost-effective compared to traditional acoustic materials, which supports its broader application in industry. The acoustic potential of the granulate stems from its porous structure and the possibility to adjust its fraction, thickness, and compaction.

Our article presents a significant contribution to the scientific literature by presenting a comprehensive experimental analysis of the acoustic properties of recycled rubber crumb in various forms and configurations. This includes the development of a patented measuring device. We develop regression models to predict acoustic performance, compare the effectiveness of recycled rubber with that of commercial materials, and provide practical, environmentally conscious recommendations. Consequently, this study expands current knowledge on the use of recycled materials in acoustics, supplementing existing research with new data, predictive models, and innovative methodologies.

This study examines the acoustic properties of recycled rubber crumb in two forms: loose granular and compact specimens. The objective is to compare their sound absorption capabilities and evaluate their potential for soundproofing applications. Measurements were made using an impedance tube, and a cartridge was developed for granular materials, enabling extended measurement options for these materials. Experimental samples of recycled rubber crumb were prepared with varying thicknesses (2 cm, 4.5 cm, 7 cm) and fraction sizes (≤1 mm, 1–2 mm, 2–3 mm, 3–4 mm). The samples were compacted under pressures of 250 kPa, 500 kPa, and 750 kPa using a filling device within test cartridges (inner diameter: 4.9 cm). For comparison, compact samples were cut from commercially available panels in the same thicknesses (2 cm, 4.5 cm, 7 cm) with a diameter of 5.9 cm. These panels are costly to produce, whereas recycled crumb offers a cost-effective and eco-friendly alternative. The research focused on analyzing how fraction size, thickness, and compaction pressure influence the transmission loss parameter (TL, in dB).

## 2. Materials and Methods

Acoustic materials, as noted earlier, are widely used in industrial and everyday environments to reduce noise and improve acoustic conditions. They are increasingly applied in homes, offices, and production facilities. In construction, these materials serve as inter-floor layers, partitions, or insulation boards to enhance sound insulation and mitigate impact noise. In transportation infrastructure, they line tunnels, airports, and platforms to dampen traffic noise. Industrial applications include anti-vibration membranes and insulation panels to reduce structural vibration and noise transmission. In healthcare settings, they improve acoustic conditions in clinics and operating rooms [23,24].

In the context of environmental sustainability and material recycling, rubber recyclate (crumb) represents a viable alternative for acoustic applications. Shredding waste rubber into granules (0.5–4 mm grain size) creates a heterogeneous porous structure with a sound absorption coefficient of α ≥ 0.6 in medium and high frequencies (above 1000 Hz) [25]. Pressing or binding the crumb with polymer adhesives produces compact samples that, due to their homogeneous composition and optimized thickness, provide consistent noise attenuation even at low frequencies [23]. These compact rubber modules achieve transmission loss (TL) levels comparable to conventional synthetic foams while offering superior resistance to mechanical wear and moisture [25,26,27]. Key parameters—such as rubber crumb grain size, binder quantity, and pressed sample geometry—can be tailored to specific application requirements, enabling broad use in construction, transportation, and industry. Modular production processes support customized solutions, ranging from thin acoustic inserts for partitions to robust noise barriers and flooring systems. Implementing rubber recyclate and its compact variants not only advances environmental sustainability through recycling but also delivers cost-effective, high-performance acoustic solutions across practical applications.

The obtained data were then statistically processed using several software tools, namely Minitab in version 18 (regression analysis), Python version 3.10.11 (using the Pandas version 2.2.3 and Seaborn libraries version 0.13.2), program R version 4.4.3 (Stat package), and OriginPro 2019b for the graphical processing of the results. Results include regression models and visualizations illustrating the dependence of transmission loss on the tested variables.

### 2.1. Development and Construction of Test Cartridges for the Experimental Evaluation of Acoustic Properties of Granular Materials

The development of test cartridges for measuring acoustic properties was necessary for a number of reasons, and it is one of the key elements of this research. Standard impedance tubes are primarily designed for testing compact samples and are therefore not suitable for directly evaluating loose granular materials. To overcome this limitation, we designed a new type of cartridge that allows for the safe and accurate placement of granular samples within the measurement system.

The main innovation of this cartridge is its versatility; it enables the testing of not only granular materials of various fractions but also pressed, compact samples. The cartridge’s design allows for the creation of sandwich structures (e.g., granular material–air gap–compact layer–granulate), while the filling module (compression device) ensures the regulation of the density of the material (200–800 kg/m^3^) at operating pressures up to 750 kPa. In this way, it is possible to accurately simulate real layering in structures and analyze the effect of compaction on the acoustic properties of materials such as glass wool, rubber crumb, or textile fibers. The test cartridge thereby not only serves as a measuring element but also functions as an integration element of the filling system.

**Figure 1:**
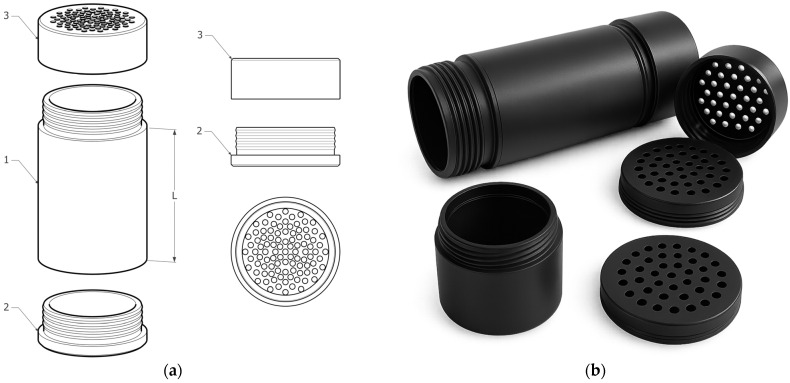
Test cartridge with interchangeable perforated parts and threaded cap. (**a**) Technical drawing: 1—Cartridge body (intended for insertion of test materials), 2—Lower perforated bottom/sieve (retains the material inside the cartridge), 3—Upper perforated lid/sieve (and also the bottom prevents the spilling of material), L—length of the cartridge, which enables adaptation to different lengths of test samples; (**b**) 3D model of the test cartridge and its individual parts.

The cartridge body is made of aluminum alloy (Al 6082-T6) with a wall thickness of 5 mm. The perforated screens are made from 1.4301 stainless steel, with 3 mm diameter holes spaced 2 mm apart, which prevents granular material from falling through. The inner diameter of the cartridge is 49 mm. The sample height is variable—20, 50, 75, 100, and 120 mm—enabling different thicknesses to be tested without the need to replace the entire cartridge. Figure 1 shows the cartridge design, featuring a threaded connection between the cartridge body and the two perforated sections (bottom and lid), which enables easy filling and emptying of the material to be tested.

The validation procedure included verifying the uniformity of the acoustic field using reference measurements with polyurethane foam for calibration (250 kg/m^3^, α = 0.95 ± 0.03). This confirmed that the cartridge does not create unwanted reflections or distort the wave field in the measurement area. Sample compression was controlled by pressing the samples at 5 kPa, 10 kPa, and 20 kPa, while monitoring changes in both thickness and volumetric density of the material.

The main advantages include the following:

1. The proposed design allows for the measurement of both granular and compact samples without the need to modify the entire measurement setup.
2. The mechanism enables fast and precise adjustment of sample thickness with high repeatability, without interfering with the measurement equipment.
3. The integrated pressing and filling system with adjustable pressure enables regulation of the density (kg/m^3^) of the material to be examined.

Because of these properties, it is possible to easily investigate the effect of thickness and pressure on transmission loss (TL, dB). Additionally, the modular design of the cartridge ensures that a single component can function as both a test and filling module.

The utility model of the cartridge was registered by the Industrial Property Office of the Slovak Republic under number 9662, which confirms the originality and practical usability of this solution.

### 2.2. Semi-Automated Equipment for Filling and Compacting Granular Materials in Test Cartridges

**Figure 2:**
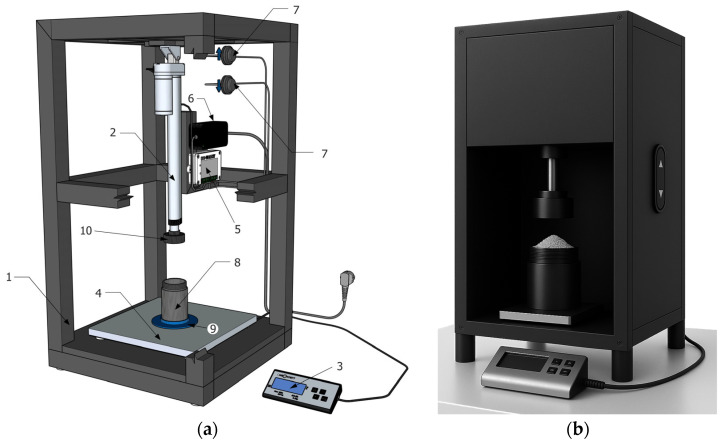
Laboratory filling and compaction apparatus with digital control. (**a**) Technical drawing: 1—Equipment frame, 2—Linear electric piston, 3—Control panel, 4—Integrated scale, 5—Control unit, 6—Power supply, 7—Vertical movement buttons (up/down), 8—Test cartridge, 9—Granular material holder, 10—Pressure mechanism; (**b**) 3D model of the device and its individual parts.

A semi-automated device was developed for filling test cartridges with granular materials (Figure 2), significantly expanding the capabilities for measuring the acoustic properties of recycled granular materials from end-of-life vehicles. This equipment allows for the application of various pressures to the granular material, enabling controlled sample compaction. As a result, it is possible to analyze the influence of compaction and thickness on the acoustic properties of the tested samples, specifically transmission loss.

The device is constructed of high-quality aluminum, while the protective cover is made of a robust steel sheet, which ensures high durability and a long service life. The overall dimensions of the device are 410 mm × 754 mm × 414 mm and its weight is 15 kg, which enables easy handling even in laboratory conditions. When measuring the noise at a distance of 30 cm from the device, a value of 50 dB was recorded, which means that the device works relatively quietly and does not disturb the surroundings. The function of the device is to press the granular material inside the cartridge. The procedure is as follows:

1. Preparation: The required amount of recycled material is poured into the cartridge, ensuring that it is evenly distributed throughout the cartridge volume. The cartridge is then inserted into the centering ring, which guarantees accurate and repeatable cartridge placement in the device, minimizing the risk of improper pressing and ensuring consistency of results in further sample processing.
2. Pressing: Using a linear piston driven by an electric motor, the material is compressed to the desired pressure. The compression force is displayed on the control panel, and the device allows pressure settings from 250 to 750 kPa in 50 kPa increments. This range provides precise and repeatable compaction tailored to the specific application requirements.
3. Closure: After compaction, the lid is securely screwed onto the cartridge, maintaining the stability and integrity of the sample during handling and subsequent measurement. This step is important to maintain the desired degree of compaction and to prevent undesirable changes in the properties of the sample before it is inserted into the impedance tube.
4. Preparation for measurement: The compacted sample is then inserted into an impedance tube for accurate measurement of its acoustic properties, specifically transmission loss (TL, dB). Measurements follow the STN EN ISO 10534-2 standard, typically using the transfer function method, which employs two or more microphones to analyze the standing wave within the tube. The test cartridge prevents material spillage and ensures measurement repeatability. Results are processed using VA-Lab4 software, providing data on the acoustic behavior of the recycled material across the frequency range of 100 Hz to 2.5 kHz.

The device is designed for efficient and repeatable pressing granular or crushed/ground materials, which is essential for accurate acoustic and material analyses. Its robust aluminum and sheet steel construction ensures a long service life and reliability even with frequent use. With its intuitive operation, the ability to precisely adjust the pressure over a wide range and the automated electric drive, the device is ideal for laboratories, research facilities and industrial operations where high accuracy and repeatability of sample preparation are required.

The semi-automated equipment for filling and compacting granular materials in test cartridges has received a utility model approved by the Industrial Property Office of the Slovak Republic under registration number 9665. This entry officially confirms the original design and practical usability of the device in laboratory and industrial conditions.

### 2.3. Characteristics of the Material Samples Tested

The recycled rubber crumb in various fractions was supplied for research purposes by a company specializing in the processing of waste tires and the production of rubber granulate. This material is widely used in the construction industry, particularly as an additive in asphalt mixtures at proportions of 15–22%, enhancing the flexibility and durability of road surfaces. Additionally, recycled rubber crumb is commonly used as infill for sports fields, contributing to shock absorption and increased surface safety. The supplier is one of the largest producers of rubber granulate in Slovakia, with operations encompassing comprehensive waste management, including recycling and recovery of secondary raw materials.

Granular samples were obtained from waste tires by means of a mechanical crushing process. The obtained rubber crumb was sieved through four circular sieves under laboratory conditions to obtain the required size fractions (up to 1 mm, 1–2 mm, 2–3 mm, 3–4 mm) for the experimental samples. This procedure ensured accurate control of the granulometry of the material, which was important for evaluating the acoustic properties of individual fractions. The compact panels were made of granulate fraction 0–4 mm using the CONIPUR 372/1 polyurethane binder (CONICA AG, Schaffhausen, Switzerland), which provides high mechanical resistance and stability at temperatures up to 150 °C. The compaction process took place at a temperature of 80–100 °C and a pressure of 2–3 MPa for 30–60 min, which ensured a homogeneous structure without internal defects. Experimental samples of granular crumb were prepared in three thicknesses (2 cm, 4.5 cm, 7 cm), while compact samples were waterjet cut to equal thicknesses with a diameter of 5.9 cm, ensuring dimensional accuracy and material integrity without thermally affecting the structure. The samples were made in two basic versions, namely loose granular (p = 0 kPa) and compacted under pressure (250 kPa, 500 kPa, 750 kPa) in test cartridges with an inner diameter (4.9 cm) using a device for filling them.

This study aims to compare the acoustic parameters between granular and compact forms of recycled rubber, using an impedance tube measurement methodology. The choice of materials was influenced by the pursuit of environmental sustainability, the easy availability of raw materials, and the expected acoustic properties, which are also confirmed by relevant scientific studies [25,28,29,30,31]. The properties of individual tested samples are listed in Table 1.

Table 1 shows the average values of the specific mass (ρ, kg/m^3^) of recycled rubber granulate, depending on the size fraction, which were determined by laboratory measurement of loose granular material without external compaction pressure. The relative deviations of measurements for granular materials do not exceed 5%, which corresponds to the variability of experimental conditions, such as the heterogeneity of the sample and the accuracy of the measuring instruments used.

### 2.4. Measurement of Transmission Loss (TL) Using an Impedance Tube

Several measurement methods have been developed to determine transmission loss (TL), with one of the most widely used being impedance tube measurement. The impedance tube has an advantage over other measurement methods due to its compactness and the ability to obtain results quickly. For example, compared to an echo chamber, it requires significantly smaller samples of the material to be tested.

**Figure 3:**
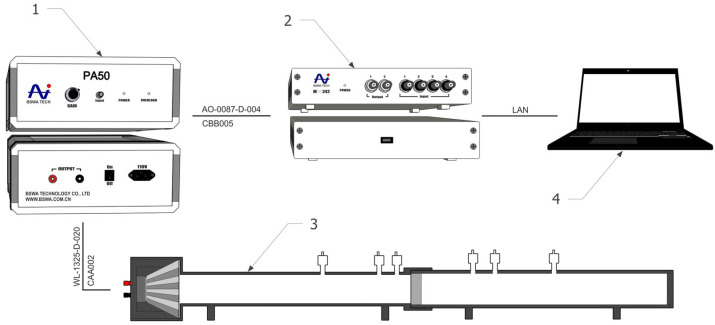
Connection diagram for measuring equipment [32]. Explanatory note: 1—PA50 power amplifier (50 W) for driving the speaker in the impedance tube; 2—MC3242 data acquisition analyzer with 4 ICP input channels and 2 output channels (0~20 kHz); 3—Impedance tube BSWA SW433; 4—PC with Va-Lab4 software.

In this study, transmission loss of recycled rubber materials was measured using a BSWA SW433 impedance tube (BSWA Technology Co., Ltd., Beijing, China) in a four-microphone configuration, with a length of 500 mm, an inner diameter of 60 mm, and a frequency range of 100–2500 Hz (see Figure 3). The measurement setup also included a 4-channel MC3242 analyzer (BSWA Technology Co., Ltd., Beijing, China) (0–20 kHz), a PA50 amplifier (BSWA Technology Co., Ltd., Beijing, China) (50 W) to power the tube’s speaker, a computer with VA-Lab4 software version 6.0, the necessary wiring, and the impedance tube itself. Transmission loss measurement was performed using the transformation function method in accordance with STN EN ISO 10534-2 (730537) [32,33]. Five repeated measurements were taken for each sample to ensure statistical reliability. To assess the impact of sample thickness, particle size fraction (rubber granules versus compact samples), and compaction pressure on transmission loss (TL, dB), experimental samples were prepared with standardized parameters, as described in detail in Section 2.3.

Key parameters of sample preparation included the following:

1. Compaction of samples using calibrated equipment with precisely set pressure (for more details, see Section 2.2).
2. Sorting rubber crumb particles by sieving through sieves with standardized holes.
3. Stabilization of samples in an air-conditioned environment prior to measurement.

This procedure ensured a high degree of reproducibility and relevance of the experimental data obtained.

Temperature (22.5 ± 1 °C) and relative humidity (48 ± 5% RH) values were monitored throughout the experiment. An atmospheric pressure of 986.07 hPa indicated a stable laboratory environment suitable for reproducible measurements.

Transmission loss was determined as a decibel (dB) value that expresses the ratio between the intensity of the sound falling on the front of the sample and the intensity of the sound transmitted through the test material. This parameter enabled us to assess how effectively the material dampens sound—the higher the transmission loss value, the better the damping properties of the material. The measurement was performed in a BSWA SW433 impedance tube, where we transmitted a broadband stationary signal PA50 (50 W) to the material sample. Using an array of microphones, we recorded the incident and reflected component of the sound on the front of the sample, and also the transmitted and reflected component on the back of the sample. This approach enabled precise determination of the proportion of sound energy transmitted, reflected, or absorbed by the material.

During the measurement, we monitored the influence of several factors: the frequency of mechanical oscillation, the thickness of the material, its internal structure (thickening under pressure—reducing porosity and increasing density), and the temperature of the environment.

We calculated the transmission loss (*TL*) according to the following equation:

(1) $${TL=10\log}_{10}\left|\frac{W_{i}}{W_{t}}\right|,$$

where *W_i_* is the energy of the incident sound wave that comes towards the test sample, *W_t_* is the energy of the transmitted sound wave that departs from the test sample.

### 2.5. Methods of Data Analysis and Processing

#### 2.5.1. Transfer Matrix Method

For the assessment and comparison of the determined transmission loss, terms describing the acoustic properties of the examined samples will be defined: transmission loss (*TL_f_*), transmission coefficient (*τ_f_*), average transmission coefficient (*τ*_avg_), relative transmission ratio (*RTR_f_*), average transmission loss (*TL*_avg_).

Transmission loss (TL, dB) is defined as the logarithm of the ratio of the incident and transmitted sound wave (power or intensity); see Equation (1).

Equation (2) for transmission loss, which is equivalently expressed by transmission coefficient (*τ_f_*) at frequency *f*, can be written as follows:

(2) $$TL_{f}=-10\log_{10}\left(\tau_{f}\right),$$

where *τ_f_* is the proportion of the transmission power.

To correctly add our average decibel (dB) values, each value must first be converted from dB to linear values. This is because a decibel is a logarithmic unit expressing the ratio of two values (such as power or intensity) and therefore cannot be treated directly as a regular numerical value.

From each measured value of the transmission loss at frequency *f*, we calculate the linear power transmission coefficient according to Equation (3):

(3) $$\tau_{f}=10^{-\frac{TL_{f}}{10}},$$

where *TL_f_* is the respective transmission loss at frequency *f*.

The power transmission coefficient expressed as a percentage *τ_f_* × 100% indicates the ratio of the sound power transmitted by the material or structure to the total incident power across the entire frequency band (in our case 100–2500 Hz). A higher transmission percentage means better transmission through the material and less power loss.

Interpretation of the value of *τ_f_*:

1. If *τ_f_* × 100% → 100%, the output power equals the input power, meaning there is no attenuation (0 dB attenuation).
2. If *τ_f_* × 100 % → 50%, about 50% of the input power is transmitted, which corresponds to a transmission loss of approximately 3 dB.
3. If *τ_f_* × 100% → 10%, about 10% of the input power is transmitted, which corresponds to a transmission loss of approximately 10 dB.
4. If *τ_f_* × 100% → 0%, almost no input power is transmitted, indicating nearly complete attenuation.

The value of 1/*τ_f_* expresses the ratio of input power to output power; in other words, it indicates how much greater the input power is compared to the transmitted (output) power. Next, we calculate the energy (linear) average of the transmission coefficient *τ*_avg_ for the frequency band 100–2500 Hz (see Equation (4)).

(4) $$\tau_{\mathrm{avg}}=\frac{1}{n}\sum_{i=1}^{n}\tau_{f},$$

where *n* is the number of frequency bands.

To calculate the average transmission loss in decibels, the following Equation (5) is used:

(5) $$TL_{\mathrm{avg}}=-10\log_{10}\left(\tau_{\mathrm{avg}}\right).$$

This formula allows converting the average transmission coefficient τ*_f_* into the corresponding transmission loss in decibels.

When comparing samples, *A* and *B*, the transmission coefficient of power of both samples will be evaluated using the relative transmission ratio (*RTR_f_*), for which the following Equation (6) applies:

(6) $$RTR_{f}=\frac{\tau_{f,vzorkaA}}{\tau_{f,vzorkaB}},$$

where *τ_f,samplesA_* is the power transmission coefficient of sample *A* at frequency *f* a *τ_f,samplesB_* is the power transmission coefficient of sample *A* at the same frequency. The power transmission ratio indicates how many times the power transmission coefficient of one sample is greater or smaller than that of another. Interpretation of the *RTR_f_* value:

1. If *RTR_f_* > 1, sample A transmits more input power at the given frequency than sample B. This indicates sample A has lower transmission loss (less power loss) compared to sample B.
2. If *RTR_f_* < 1, sample A transmits less input power at the given frequency than sample B. This means sample A has greater transmission loss (more power loss) compared to sample B.
3. If *RTR_f_* ≈ 1, both samples transmit nearly the same amount of input power at the given frequency, indicating similar transmission loss between the two.

#### 2.5.2. Regression Analysis

To determine the relationship between variables, we consider variable *Y*, which represents the dependent (output) variable, and the variables *X_1_*,*X_2_*,…,*X_k_*, which represent *k*-independent (input) variables. The relationship between the dependent variable *Y* and independent variables *X_i_* (where *i* = 1,…,*k*) can be described using the following multiple linear regression model (see Equation (7)):

(7) $$Y=\beta_{0}+\sum_{i=1}^{k}\beta_{i}X_{i}+\varepsilon ,$$

where *β_0_* is the intercept, *β_1_*,*β_2_*,…,*β_k_* are the regression coefficients corresponding to each independent variable, and ε is the error term, representing the variability not explained by the model.

The statistical significance of the entire regression model can be tested using the F-test. To verify the statistical significance of individual parameters of the regression model, tests of statistical significance of regression coefficients, most often *t*-tests, are used. When testing statistical hypotheses, the decision to reject or accept the null hypothesis is implemented based on the *p*-value. If the *p*-value is less than the established significance level *α*, we reject the null hypothesis in favor of the alternative hypothesis. If the *p*-value value is less than the established significance level *α*, we reject the null hypothesis in favor of the alternative hypothesis. If the *p*-value is equal to or greater than the chosen significance level of α, then we do not reject the null hypothesis.

The strength of the dependence of the variable *Y* on the effect on the independent variables is expressed by a multiple coefficient of determination *R*^2^, which has values in the interval ⟨0; 1⟩. The closer the value of *R*^2^ is to 1, the closer the relationship between the dependent and the independent variables, and the model explains more of the dependent variable’s variability. Because variables in regression analysis can be expressed in different units and scales, standardized regression coefficients β^*^_i_, where *i* = 1,…,*k*, are often used to compare the relative importance of individual input variables. The standardized regression coefficient β^*^_i_ expresses how many standard deviations the output variable *Y* changes when the standardized deviation of the independent variable *X_i_* changes by one unit, assuming that the other input variables remain constant. The higher the absolute value of the coefficient β^*^_i_, the stronger the influence of the independent variable in question on the output variable.

## 3. Results

In this section, we present a comprehensive analysis of the acoustic properties of recycled rubber crumb, with a particular emphasis on sound transmission loss. The study investigates how various sample parameters affect the material’s ability to dampen sound, focusing on four main variables: rubber crumb fraction (ranging from fine particles of 1 mm to coarse particles of 3–4 mm); sample thickness (2 cm, 4.5 cm, and 7 cm); compression pressure of the material (0 kPa—loose granular material, 250, 500, and 750 kPa—granular material); sound frequency (ranging from 100 Hz to 2500 Hz).

Methodologically, we compare the sound transmission loss and transmission coefficient of compacted samples with those of loose granular rubber crumb under various configurations of these parameters. For each sample, under different configurations of the specified parameters (pressure, thickness, and fraction), five repeated measurements of transmission loss were performed. To enable comparison of the observed properties, the average transmission loss was calculated at each frequency within the considered frequency range. The results provide a systematic evaluation of the measured values, aiming to identify the optimal properties of recycled rubber crumb for maximizing its acoustic insulation capabilities. The insights gained from this research have the potential to support more efficient use of recycled rubber materials in acoustic applications, thereby promoting environmentally friendly waste management while enhancing sound insulation in buildings, industrial plants, and other environments where noise reduction is essential.

### 3.1. Defining Variables

Recycled tire-derived rubber crumb demonstrates distinctive acoustic properties that make it suitable for sound insulation and absorption applications. Studies indicate its effectiveness in noise reduction, attributed to its porous internal structure [34,35,36,37].

Table 2 provides a systematic overview of the material’s properties, experimental parameters, and variables characterizing the analyzed rubber crumb samples.

### 3.2. Acoustic Performance Evaluation of Recycled Rubber Crumb

#### 3.2.1. Transmission Loss and Transmission Coefficient Analysis

**Figure 4:**
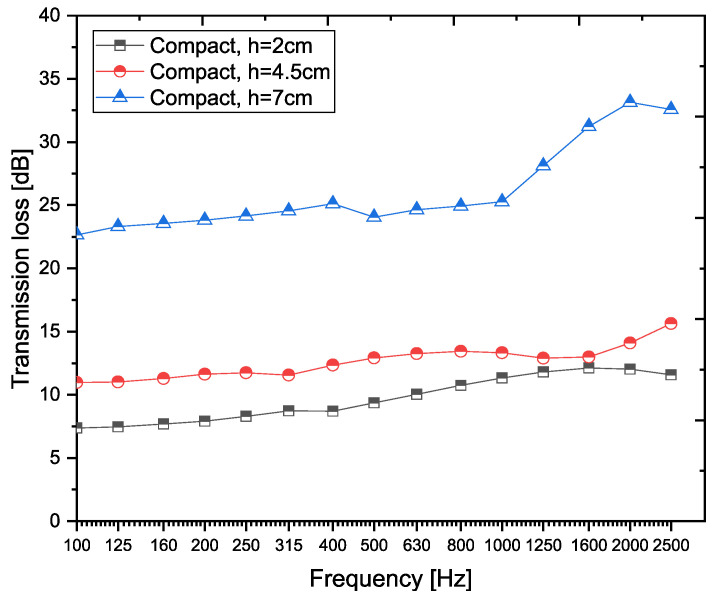
Transmission loss (TL) values compact recycled rubber granulate (RRC) samples at different thicknesses across frequencies.

The compact recycled rubber crumb (RRC) was produced in three different thicknesses: 2 cm, 4.5 cm, and 7 cm. The best transmission loss values (in dB) were achieved by the sample with a thickness of 7 cm (TL_C,7_). The highest values were measured in the 7 cm sample within the frequency range of 1600 Hz to 2500 Hz, with the maximum transmission loss, TL_C,7_ = 33.13 dB, recorded at a frequency of 2000 Hz (see Figure 4).

**Figure 5:**
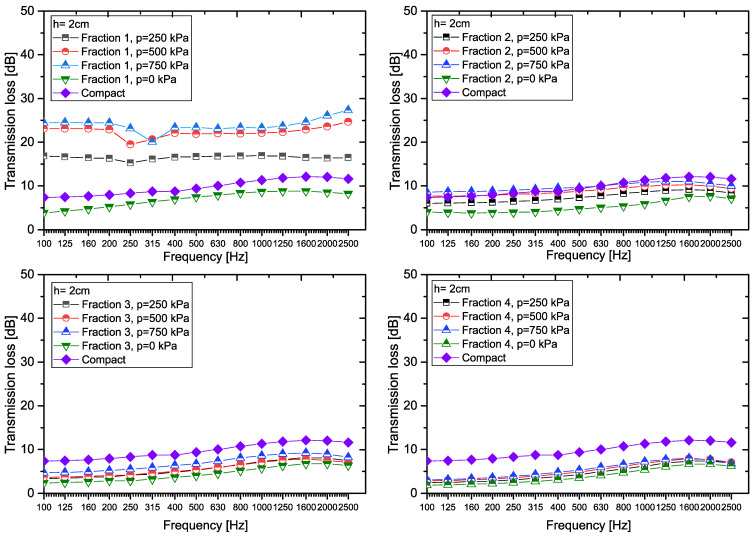
Graphical representation of the measured TL values for RRC samples (h = 2 cm).

A graphical representation of the transmission loss values for samples of granular recycled rubber crumb, including the effects of particle size fraction, compaction pressure, and thickness, is presented in Figure 5. To enhance the informative value, the graphs also include comparative data for compact recycled rubber crumb at equivalent sample thicknesses, enabling direct analysis of the differences between granular and compact forms of the material.

For the 2 cm thick sample (Figure 5), the highest transmission loss (TL, in dB) values were observed in *Fraction 1* under compaction pressures of 500 kPa and 750 kPa. Conversely, the lowest TL values occurred in *Fraction 4*, with minimal variation across pressure levels. Notably, *Fractions 3* and *4* (particle sizes 2–4 mm) exhibited nearly identical TL values, likely due to their small particle size differences and identical material composition.

Compared to the transmission loss values for *Compact RRC* and *Fraction 1*, the following applies: TL_F1,2,0_ < TL_C,2_ < TL_F1,2,250_ < TL_F1,2,500_ < TL_F1,2,750_. For *Fraction 2* it applies that TL_F2,2,0_ < TL_F2,2,250_< TL_C,2_ < TL_F2,2,500_ < TL_F2,2,750_, and for *Fraction 3*, or *Fraction 4* it applies that TL_F3,2,0_ < TL_F3,2,250_ < TL_F3,2,500_ < TL_F3,2,750_ < TL_C,2_, resp. TL_F4,2,0_ < TL_F4,2,250_ < TL_F4,2,500_ < TL_F4,2,750_ < TL_C,2_.

Table 3 shows the transmission coefficient *τ_f_* at frequency *f*, expressed as a percentage, for samples with a thickness of 2 cm at various particle size fractions and compaction pressures across the entire frequency range, including data for *Compact RRC* of the same thickness. To compare the efficiency of the individual samples, the table also presents the average linear transmission coefficient *τ*_avg_ (in %) and the average transmission loss *TL*_avg_ (in dB), both calculated over the full frequency spectrum.

The analysis reveals that for *Fraction 1* under a pressure of 250 kPa at a frequency of 100 Hz, only approximately 2.1% of the original sound power remains, meaning the transmission coefficient is 2.1%. In contrast, for *Fraction 4* at a pressure of 0 kPa and the same frequency, as much as 64.8% of the original power remains, indicating significantly lower transmission loss compared to *Fraction 1*. *Fraction 1* samples achieve the lowest transmission coefficients at pressures of 500 kPa and 750 kPa, while *Fraction 4* samples consistently show the highest transmission coefficients. For each fraction, samples made from loose granular recycled rubber crumb (p = 0 kPa) exhibit the highest transmission coefficients. In most cases, except for *Fraction 1* at pressures of 250 kPa and 500 kPa, the highest transmission coefficient is recorded at the lowest frequency (100 Hz), with values generally decreasing as frequency increases. The most pronounced differences between the lowest and highest frequencies are observed in *Fraction 3* and *Fraction 4*. For the *Fraction 2*, *Fraction 3*, and *Fraction 4* samples, the transmission coefficient decreases up to a frequency of 1000 Hz, after which it increases, resulting in reduced attenuation effects at higher frequencies.

The experimental results indicate that, to achieve maximum sound insulation, Fraction 1 should be used under pressure (at or above 500 kPa), as this configuration provides the greatest attenuation effect across a wide frequency spectrum. For specialized applications that require the suppression of sound waves within a specific frequency range, combinations involving larger fractions with variable pressure profiles can also be effective alternatives.

**Figure 6:**
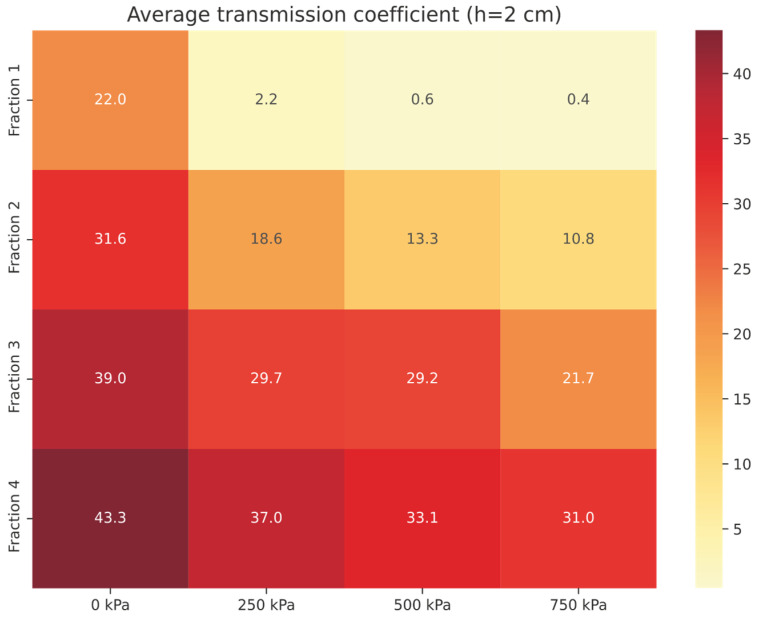
Average transmission coefficient *τ*_avg_ of granular RRC (h = 2 cm).

For a 2 cm thick *Compact RRC* sample, the average transmission coefficient *τ*_avg_ (in%) for the 100–2500 Hz frequency band was determined to be 11.62% (see Table 3). In contrast, graphical analysis (see Figure 6) shows the average transmission coefficient in the same frequency range for granular *RRC* with a thickness of 2 cm, across all investigated fractions.

In the case of sample *Fraction 1* (thickness 2 cm, pressure 250 kPa), the average transmission coefficient *τ*_avg_ = 2.2%, which means that the material allows only 2.2% of the incident energy to pass through, and the rest (97.7%) is absorbed or lost due to absorption or reflection (Figure 6). In comparison, for the same fraction with zero pressure, *τ*_avg_ increases to 22%, demonstrating a significant effect of the compaction of the sample under pressure on the attenuation properties of the material. The highest *τ*_avg_ values are shown by *Fraction 4* (31.0–43.3%) and *Fraction 3* (21.2–39.3%) samples, with ranges reflecting the dependence on the applied pressure. These results confirm that higher *Fractions (3–4)* show lower attenuation in the given frequency band.

**Figure 7:**
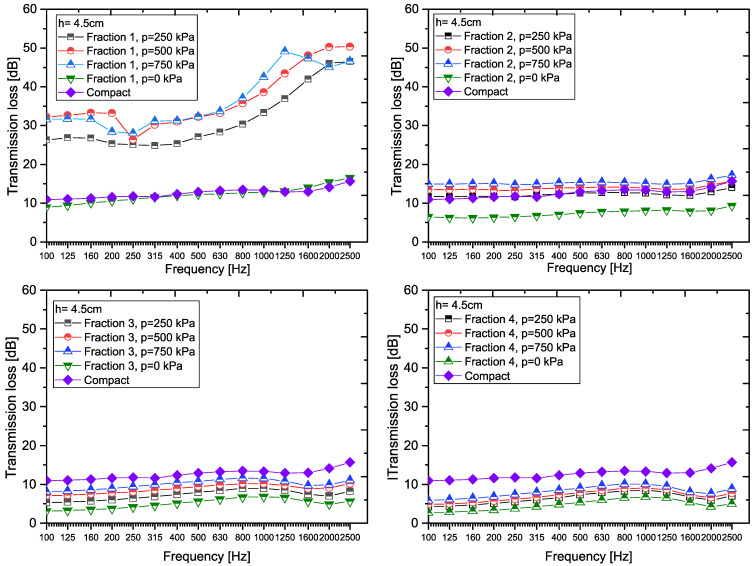
Graphical representation of the measured TL values for RRC samples (h = 4.5 cm).

For samples with a thickness of 4.5 cm, *Fraction 1* samples achieved the highest transmission loss values at pressures of 500 kPa and 750 kPa, while at approximately 400 Hz, the transmission loss values began to increase (see Figure 7). Also in this case, the transmission loss values for *Fraction 3* and *Fraction 4* samples are very similar, which indicates comparable acoustic properties of these fractions regardless of the increased sample thickness.

When comparing the transmission loss values for all samples with a thickness of 4.5 cm, the following trends can be observed. For *Fraction 1*, the relationship is as follows: TL_F1,4.5,0_< TL_C,4.5_ < TL_F1,4.5,250_< TL_F1,4.5,500_ < TL_F1,4.5,750_. For *Fraction 2* it applies that TL_F2,4.5,0_ < TL_C,4.5_ < TL_F2,4.5,250_ < TL_F2 4.5,500_ < TL_F2,4.5,750_, and for *Fraction 3*, or *Fraction 4* it applies that TL_F3,4.5,0_ < TL_F3,4.5,250_ < TL_F3,4.5,500_ < TL_F3,4.5,750_ < TL_C,4.5_, or TL_F4,4.5,0_ < TL_F4,4.5,250_ < TL_F4,4.5,500_ < TL_F4,4.5,750_ < TL_C,4.5_.

Table 4 shows the transmission coefficient *τ_f_* (in %) for samples with a thickness of 4.5 cm at different fractions and pressures throughout the frequency band, including the *Compact RRC* of the same thickness. To compare the efficiency of individual samples, the table also shows the average linear transmission coefficient *τ*_avg_ (in %) and the average transmission loss *TL*_avg_ (in dB), calculated for the entire frequency spectrum.

Also in this case, *Fraction 1* samples show the lowest transmission coefficient at pressures of 500 kPa and 750 kPa, which means that these samples achieve the highest transmission loss. On the contrary, the highest transmission coefficient (i.e., the lowest transmission loss) was found in *Fraction 4* samples. For all fractions, samples made from loose granular (p = 0 kPa) recycled rubber crumb exhibit the highest transmission coefficient, indicating a lower ability to dampen acoustic energy compared to compacted samples.

In all cases, the highest transmission coefficient occurs at the lowest frequency, i.e., at 100 Hz. For *Fraction 2*, *Fraction 3*, and *Fraction 4* samples, the transmission coefficient decreases with increasing frequency up to 1000 Hz. Above this frequency, there is a resurgence of the transmission coefficient, which means a decrease in attenuation effects in this part of the frequency spectrum. These results confirm the importance of the combination of fraction size, applied pressure, and material thickness for optimizing the acoustic properties of the tested samples.

**Figure 8:**
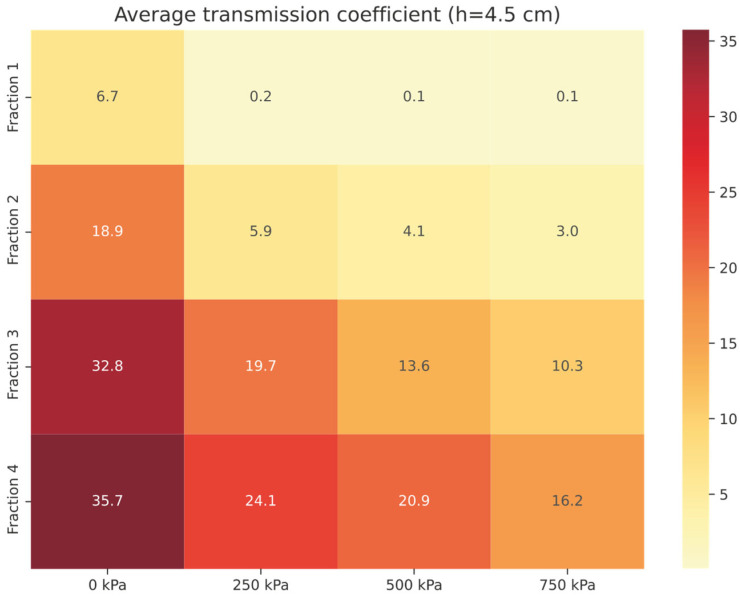
Average transmission coefficient *τ*_avg_ of granular RRC (h = 4.5 cm).

The average transmission coefficient *τ*_avg_ (in %) was determined to be 5.70 % in this case for a *Compact RRC* sample with a thickness of 4.5 cm (see Table 4). Figure 8 shows a graphical analysis of *τ*_avg_ (in %) for a granular RRC sample of the same thickness (h = 4.5 cm).

For *Fraction 1* (4.5 cm thickness, excluding loose granular RRC, p = 0 kPa), the average transmission coefficient (*τ*_avg_) was measured at 0.1%, reflecting exceptional sound attenuation. The highest values of the average transmission coefficient were again recorded for the samples *Fraction 3* and *Fraction 4*—loose granular RRC pressure 0 kPa.

**Figure 9:**
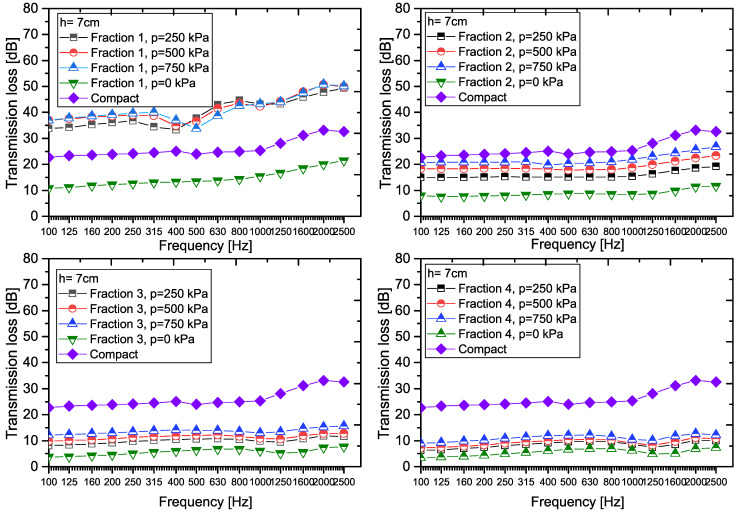
Graphical representation of the measured TL values for RRC samples (h = 7 cm).

Experimental results confirm that the *Fraction 1* sample at the applied pressure of 750 kPa shows the highest average transmission loss *TL*_avg_, which ranks it among the materials with the most effective acoustic insulation properties (see Figure 9). In comparison, *Fraction 4* at pressure 0 kPa achieves the lowest damping efficiency, which means a low ability to suppress the propagation of sound energy.

When comparing the transmission loss values for all samples with a thickness of 7 cm, the following trends can be observed. For *Fraction 1*, the relationship is as follows: TL_F1,7,0_ < TL_C,7_ < TL_F1,7,250_ < TL_F1,7,500_ < TL_F1,7,750_. For *Fraction 2* it applies that TL_F2,7,0_ < TL_F2,7,250_ < TL_C,7_ < TL_F2,7,500_ < TL_F2,7,750_, and for samples *Fraction 3*, or *Fraction 4* it applies that TL_F3,7,0_ < TL_F3,7,250_ < TL_F3,7,500_ < TL_F3,7,750_ < TL_C,7_, or TL_F4,7,0_ < TL_F4,7,250_ < TL_F4,7,500_< TL_F4,7,750_ < TL_C,7_.

Table 5 presents the transmission coefficient *τ_f_* (in %) for samples with a thickness of 7 cm at different fractions and pressures throughout the frequency band, including *Compact RRC* of the same thickness. In addition, the table also includes the average linear transmission coefficient *τ*_avg_ (in %) and the average transmission loss *TL*_avg_ (in dB). All these values provide an overview of the acoustic properties of individual samples.

*Fraction 1* samples at pressures (500–750 kPa) show an almost zero average transmission coefficient (*τ*_avg_ ≈ 0%), which ranks them among materials with excellent acoustic properties. These results confirm their effectiveness in suppressing the transmission of acoustic energy throughout the researched frequency band. Conversely, *Fraction 4* at p = 0 kPa achieves the highest values of *τ*_avg_, which indicates its reduced ability to suppress the transmission of acoustic energy. *Fraction 2* and *Compact RRC* show significantly higher attenuation at a thickness of 7 cm compared to previous experimental test conditions, which makes them effective acoustic materials. The thickness of the material is of great importance in this case. When the thickness of the sample is increased to 7 cm, the cut-off frequency is shifted to the region of low frequencies (100–500 Hz). In this band, the material begins to resonate more strongly (waves “accumulate” and amplify in it). This behavior causes the sound waves in the material to slow down and bounce off the edges, creating standing waves.

**Figure 10:**
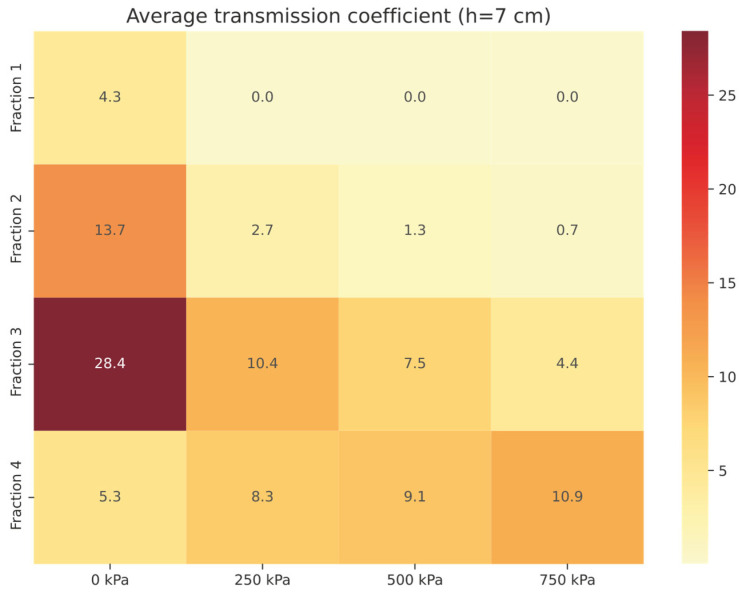
Average transmission coefficient *τ*_avg_ of granular RRC (h = 7 cm).

Experimental data have shown that the 7 cm thick *Compact RRC* has an average transmission coefficient *τ*_avg_ = 0.31% (see Table 5), confirming its almost complete impermeability to sound waves. For the loose granular (p = 0 kPa) sample with a thickness of 7 cm (Figure 10), *τ*_avg_ was the lowest compared to thicknesses of 2 and 4.5 cm, indicating the synergistic effect of the combination of compaction and sample thickness. At the same time, *Fraction 1* (except at p = 0 kPa) almost completely prevents the transmission of incident power.

These results show that optimizing sound attenuation requires a multifactorial approach—a combination of optimal particle size, sufficient material thickness, and precise pressure adjustment. The synergistic effect of these parameters determines how effectively the material suppresses the propagation of sound energy, while the interaction between the material structure and the input parameters is key to achieving the desired acoustic properties.

#### 3.2.2. Comparison of Samples Using the Relative Transmission Ratio (RTR)

For the direct comparison of the acoustic properties of compact and loose granular RRC samples at a given frequency *f*, the relative transmission ratio (RTR*_f_*) is determined by the following equation (Equation (5)):

(8) $$RTR_{f}=\frac{\tau_{f,\;compact}}{\tau_{f,\;granular}},$$

where *τ_f,compact_* represents the transmission coefficient of a *Compact RRC* sample at frequency *f*, and *τ_f,granular_* denotes the transmission coefficient of granular RRG (at pressures ranging from 0 to 750 kPa) at the same frequency *f*, with both samples having the same thickness.

**Figure 11:**
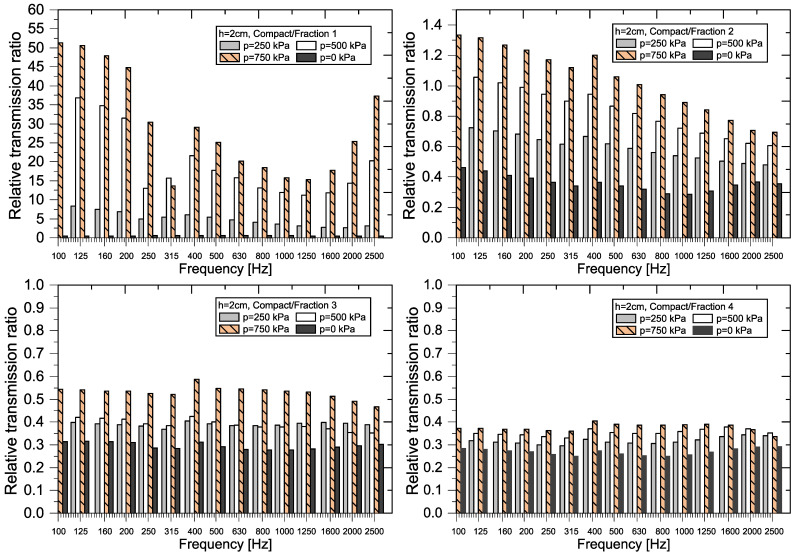
Graphical representation of RTR for RRC samples (h = 2 cm).

**Figure 12:**
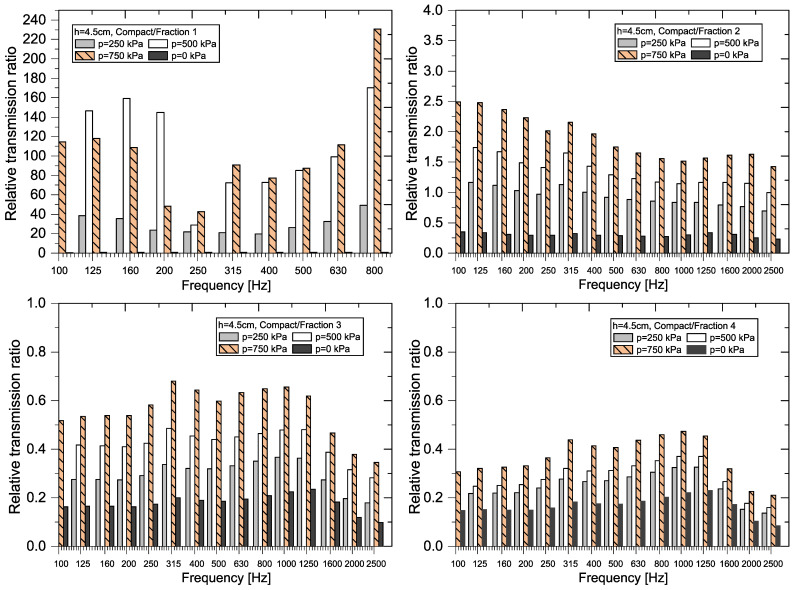
Graphical representation of RTR for RRC samples (h = 4.5 cm).

**Figure 13:**
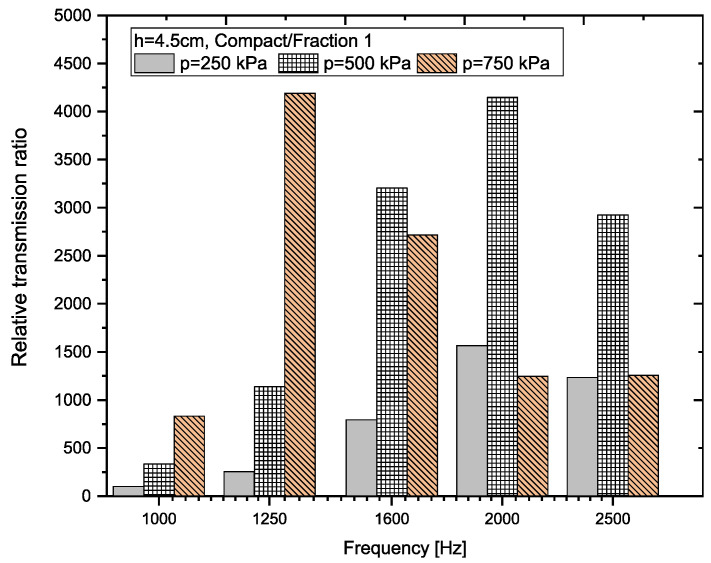
Graphical representation of RTR for RRC samples (Fraction 1, h = 4.5 cm).

**Figure 14:**
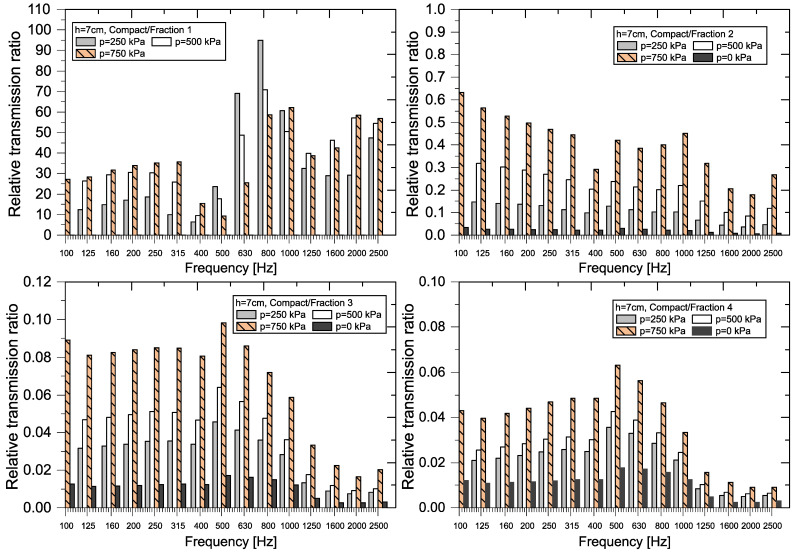
Graphical representation of RTR for RRC samples (h = 7 cm).

A graphical representation of the RTR*_f_* values (see Equation (8)) for each sample thickness and each frequency *f* is shown in Figure 11, Figure 12, Figure 13 and Figure 14.

The greatest variability of RTR values was recorded when comparing compact and granular recycled rubber crumb (p = 0–750 kPa) of *Fraction 1* (h = 2 cm). In all cases, except for the loose granular sample (p = 0 kPa), the RTR values were several times higher than 1, which means that the *Compact RRC* transmits several times more incident power than the granular crushed material. In the case of *Fraction 2*, RTR values are greater than 1 at a pressure of 750 kPa in the frequency range from 100 Hz to 800 Hz, with values approaching 1 above this limit. At a pressure of 500 kPa and in the band of 100 to 315 Hz, the transmission coefficient *τ_f_* and thereby the transmission loss of *TL_f_* of compact and granular samples of *Fraction 2* are comparable.

For *Fraction 3* and *Fraction 4* samples, the RTR values are consistently below 1, indicating that the *Compact RRC* transmits less incident power than the granular RRC (p = 0–750 kPa) at the same frequency and thickness. This corresponds to a significantly higher transmission loss for the *Compact RRC* compared to the granular RRC, with the transmitted power through the *Compact RRC* sample being, on average, 2.5 to 3.9 times lower than that of the granular RRC.

A graphical representation of the relative transmission ratio value for samples 4.5 cm thick is shown in Figure 12 and Figure 13.

The greatest variability in the relative transmission ratio (RTR) values again occurs when comparing compact and granular recycled rubber crumb (p = 0–750 kPa) of *Fraction 1* (h = 4.5 cm). In all cases, except for loose granular crumb (p = 0 kPa), the RTR values are several times higher than 1. For better clarity, Figure 13 shows ratios from 100 Hz and above. This indicates that granular RRC has a significantly better transmission loss at pressures (p = 250–750 kPa) compared to *Compact RRC*. In the case of *Fraction 2*, RTR values are greater than 1 at pressures of 500 kPa and 750 kPa, indicating a higher efficiency of granular RRC in this range. At a pressure of 250 kPa, the relative transmission ratio value is close to 1, which means that the transfer properties of both types of samples are comparable. For *Fraction 3* and *Fraction 4* samples, the RTR values are balanced and less than 1, which means that the sample from the *Compact RRC* passed through less incident power than the sample of the granular RRC at the same frequency and thickness. This corresponds to higher attenuation for the *Compact RRC* in these cases.

A graphical representation of the relative transmission ratio value for samples with a thickness of 7 cm is shown in Figure 14.

When we compare compact and loose granular recycled rubber crumb (RRC) of *Fraction 1* (h = 7 cm), we find that in all cases except for the sample with a pressure of p = 0 kPa, the granular RRC dampens noise much more effectively than the *Compact RRC*. In these cases, the values of the relative transmission ratio (RRT) are several times higher than 1, which means that the granular sample transmits significantly less sound. On the other hand, at zero pressure (loose granular crumb, p = 0 kPa), the *Compact RRC* is better, which in this case dampens the sound more effectively—RTR values are in the range of 0.05 to 0.1. Because of these extremely low values, the results for Fraction 1 at p = 0 kPa are not displayed in Figure 14.

For *Fractions 2–4*, the *Compact RRC* is always more effective in noise attenuation than a granular RRC, as the RTR values are always less than 1. Thus, the results confirm that *Fraction 1* granular RRC (p = 250–750 kPa) exhibits better acoustic properties than *Compact RRC* at all thicknesses.

### 3.3. Model Development via Regression Analysis

When analyzing the effect of selected input variables (pressure *p*, thickness *h*, frequency *Freq*) on the output variable transmission loss (TL, dB) of granular recycled rubber crumb, we will proceed using the following model:

(9) $$TL=f\left(p,h,Freq\right),$$

or for a compact recycled rubber crumb model:

(10) $$TL=f\left(h,Freq\right).$$

The best regression model for *Fraction 1* and *Fraction 2* is a model of the following form:

(11) $$TL_{granular}=\beta_{0}+\beta_{1}\cdot p+\beta_{2}\cdot h+\beta_{3}\cdot Freq+\beta_{4}\cdot p\cdot h+\varepsilon ,$$

or for *Fraction 3* and *Fraction 4*:

(12) $$TL_{granular}=\beta_{0}+\beta_{1}\cdot p+\beta_{2}\cdot h+\beta_{3}\cdot Freq+\varepsilon ,$$

where *β_0_*, *β_i_* (i = 1,2,3,4) are the parameters of the model; *p* is the pressure (kPa); *h* is the thickness of the sample (cm); *Freq* is the frequency (Hz); and *p* × *h* represents the interaction between thickness and pressure.

In the case of compact recycled rubber crumb, the best regression model has the following form:

(13) $$TL_{compact}=\beta_{0}+\beta_{1}\cdot h+\beta_{2}\cdot Freq+\varepsilon .$$

Point estimates of the regression model parameters, standardized coefficients, and their statistical significance (*p*-value) are summarized in Table 6 (see Equations (9–13)). The table also reports the multiple coefficient of determination (*R*^2^) and the overall model significance (*p*-value). All parameters and the regression models themselves are statistically significant (*p*-value < *α*), with the significance level set at *α* = 0.05.

The regression analysis showed that all input variables (pressure *p*, thickness *h*, frequency *Freq*, interaction *p* × *h*) significantly affect the transmission loss with each investigated fraction. The strength of this relationship between the transmission loss and the input variables is expressed by a multiple coefficient of determination *R*^2^. In the case of *Fraction 1*, the coefficient of determination is equal to 0.82, which means that 82.0% of the variability of the variable transmission loss can be explained by the proposed regression model. Other models have similarly high values (*Fraction 2*: *R*^2^ = 0.955 (95.5%), *Fraction 3*: *R*^2^ = 0.833 (83.8%), *Fraction 4*: *R*^2^ = 0.808 (80.8%), *Compact sample*: *R*^2^ = 0.862 (86.2%)), which represents a strong degree of dependence of the transmission loss variable on the input variables.

These results point to a strong degree of dependence of the transmission loss on the input variables and confirm that the proposed regression models are suitable for predicting the acoustic properties of recycled rubber crumb under different conditions.

The values of the parameters obtained by the regression models show that all input variables have a positive effect on the value of transmission loss. The analysis of standardized Beta coefficients (see the Beta column in Table 6) shows the following:

1. Fraction 1: the pressure variable (*p*) has the most significant effect on transmission loss, with the thickness (*h*) of the samples having a slightly smaller effect.
2. Fraction 2: the interaction between the variable *h* and the variable *p* (*p* × *h*) has the most significant influence.
3. Fraction 3: the variables pressure (*p*) and thickness (*h*) have a comparable effect on transmission loss.
4. Fraction 4: the thickness variable (*h*) has the most significant effect on transmission loss; the other two variables have a comparable effect.
5. Compact recyclable crumb: variable thickness (*h*) has by far the most significant influence on transmission loss.

These results confirm that optimizing acoustic properties depends on the appropriate selection and combination of input parameters.

## 4. Discussion and Conclusions

This paper evaluated the acoustic properties of recycled rubber crumb samples, with the main criterion being the transmission loss (TL, dB) measured by an impedance tube in accordance with international standards. The samples were prepared in two basic forms: loose granular (p = 0 kPa) and compacted under pressure (250 kPa, 500 kPa, 750 kPa). For comparison, a compact sample cut from a commercially available panel in three thicknesses (2 cm, 4.5 cm, 7 cm) was also analysed.

Based on experimental measurement of the transmission coefficients *τ_f_* (in %) and the average transmission loss *TL*_avg_ (in dB) for granular and compact recycled rubber crumb (RRC), the following key relationships between pressure *p*, thickness *h*, and frequency response *Freq* were identified:

1. Scientific and professional studies by the authors [15,26,37] focus on the analysis of the structure of individual materials and its influence on acoustic properties. The results of the research show that *Fraction 1* has the best soundproofing properties, which is reflected in the lowest value of the transmission coefficient. This trend is in line with the law of mass, according to which smaller particles form a more compact structure with a higher density, resulting in more effective sound damping. The authors [26,37] state in their publications that the size of the fraction and compactness of materials significantly affect acoustic properties, while smaller and more compact particles improve the ability of the material to dampen the propagation of sound waves. This knowledge provides a theoretical basis for optimizing the composition and structure of materials in order to achieve a higher level of sound insulation.
2. The results of experimental measurements show that thicker samples of material have much better sound insulation properties than thinner samples. This phenomenon is due to the fact that with a greater thickness of material, several factors are better applied together—in particular, the higher mass, the ability of the material to absorb vibrations (viscoelastic damping), and a more complex internal structure. These findings are also confirmed by other authors [3,4], who emphasize that it is the combination of a higher specific gravity and a more complex structure that is key to the best possible sound insulation.
3. Experimental results show that with increasing pressure, the porosity of the material decreases, and its density increases. These changes lead to a decrease in the value of the transmission coefficient *τ_f_* in all the investigated fractions, which means that the material better prevents the attenuation of sound. The most pronounced effect was observed in *Fraction 1*, where the difference in attenuation between the loose granular material and the sample exposed to pressure (p = 750 kPa) was very significant. This clearly confirms that mechanical compression, i.e., compaction of the material, significantly improves its acoustic properties. This positive effect is mainly due to the fact that compression eliminates air gaps, creating a more homogeneous and dense material structure that better absorbs and dissipates sound energy.
4. The analysis of the transmission coefficients of recycled rubber crumb shows that this material has significant potential as an effective sound insulation element, especially when using fine fractions exposed to pressure. The frequency dependence of the transmission coefficients corresponds to the theoretical assumptions of acoustics of porous materials, which is also confirmed by several scientific studies [3,15,23]. These results suggest that recycled rubber crumb may be a suitable alternative for applications aimed at improving sound insulation.
5. The analysis confirmed that the acoustic properties of recycled rubber crumb are significantly influenced by the synergistic action of the mass law, structural heterogeneity, and resonance phenomena. The increase in the thickness of the samples caused the critical frequency to shift to a lower frequency band, which has a significant impact on the effectiveness of sound insulation. For practical use, it is recommended to combine fractions of different sizes and apply a layered structure of the material, which will achieve more effective suppression of broad-spectrum noise.

If it is necessary to replace the compact recycled rubber crumb with another fraction with similar or better transmission loss values, we recommend considering the following options and approaches:

1. Fraction 3 and Fraction 4: these fractions are not recommended for use if the goal is to achieve better transmission loss, as their acoustic insulation properties are insufficient.
2. Fraction 1: Loose granular RRC (at a pressure of 0 kPa) is not suitable for use, as it does not provide sufficient attenuation. On the contrary, it is recommended to apply a pressure of 500 kPa or 700 kPa, which will significantly improve the acoustic properties of this fraction.
3. Fraction 2: Loose granular RRC (0 kPa) or granular RRC with a pressure of 250 kPa is also not suitable, because the attenuation is low in these cases. The use of a pressure of 500 kPa or 700 kPa is recommended, with a greater sample thickness further improving the transmission loss values for this fraction.

The results of the regression analysis clearly indicate a statistically significant influence of the analyzed input parameters (pressure, thickness, frequency) on the magnitude of the transmission loss. The developed regression models show a high degree of statistical significance and represent a suitable tool for predictive modeling of transmission loss behavior depending on variable input parameters for each type of sample (loose granular or compacted recycled rubber crumb).

The experimental data and results from this study have clear practical value. The findings, together with the regression models we developed, can guide the design of acoustic panels made from recycled materials, especially when estimating sound absorption performance as functions of layer thickness, particle size, and other parameters. These insights also provide a solid foundation for future research: by incorporating additional variables, subsequent models could further advance sustainable, high-performance acoustic solutions.

The morphology and particle size of the rubber crumb significantly affect its acoustic performance. Fine fractions, when highly compacted, provide a versatile solution across a broad frequency spectrum, whereas coarser fractions are better suited for targeted applications in the low-frequency range. The irregular shape and rough surface of the recycled rubber particles create variable inter-particle gaps during compaction, yielding a heterogeneous microstructure that influences transmission loss. As a result, compaction density varies: fine fractions (≤1 mm) form a relatively uniform matrix with small, evenly distributed pores, whereas coarser fractions produce larger, irregular voids. In addition, size variability within each fraction introduces localized density fluctuations, which partly account for the statistical scatter observed in transmission loss measurements.

At the same time, the experimental results show that with the right choice of fraction and applied pressure, it is possible to achieve optimal acoustic properties of recycled rubber crumb for specific applications. This underlines its potential as an environmentally friendly material for sound insulation. Recycled rubber is a sustainable alternative to traditional materials such as mineral wool, with the added advantage of being highly resistant to moisture, chemicals, and similar factors. This feature expands its potential uses, especially in industrial environments. The main advantage of loose recycled rubber materials lies in their favorable acoustic properties and environmental compatibility, as they do not contain any added chemical binders. A potential drawback, however, may be the technological challenges associated with applying these materials in noise barrier wall constructions. The authors of [38] state that the acoustic properties of materials based on recycled tires are comparable to those of standard commercially available materials used in noise barrier walls. Several authors [6,25,29] highlight the suitability of recycled materials for soundproofing applications and also point out their environmental benefits. Recycled rubber crumb thus combines technical efficiency with environmental sustainability and represents a forward-looking solution for modern acoustic applications.

## 5. Patents

The test cartridge described in this paper is the subject of patent No. 289324, “Test Cartridge for Expanding the Possibilities of Measuring Bulk Materials in an Impedance Tube”, which was granted by the Industrial Property Office of the Slovak Republic.

The laboratory device for filling and compaction with digital control described in this article is the subject of published patent application no. 50022-2022, “Device for Measured Filling of Test Cartridges with Bulk Materials”, which was granted by the Industrial Property Office of the Slovak Republic.

## Figures and Tables

**Table 1:** List and sample parameters of recycled rubber materials.

Material	Structure	Fraction (mm)	Sample Views Macro/Micro Views	Bulk Density (kg/m^3^)
Recycled rubber panel	compact	0–4	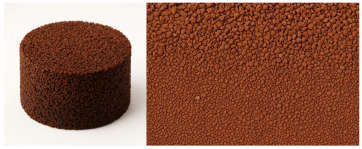	849
Recycled rubber crumb	granular	≥ 1	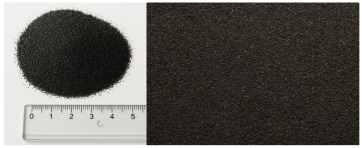	377
		1–2	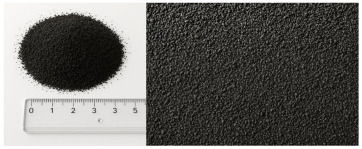	458
		2–3	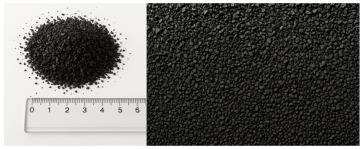	472
		3–4	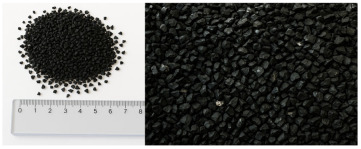	500

**Table 2:** Overview of the parameters used and properties of the examined samples.

Label	Description
F	Rubber material fraction (mm)
F1	Fraction of rubber material of size 1 mm (Fraction 1)
F2	Fraction of rubber material of size from 1–2 mm (Fraction 2)
F3	Fraction of rubber material of size from 2–3 mm (Fraction 3)
F4	Fraction of rubber material of sizes from 3–4 mm (Fraction 4)
h	Sample thickness (cm), h = 2 cm, h = 4.5 cm, h = 7 cm
p	Pressure under which the granular sample was compacted (kPa), 0 kPa (loose granular), 250 kPa, 500 kPa, 750 kPa (granular material)
Freq	Frequency (Hz), frequency band from 100 Hz to 2500 Hz
TL	Transmission loss (dB)
TL_C_	Transmission loss of compact material of recycled rubber crumb
TL_F_	Transmission loss of granular recycled rubber crumb at fraction *F*
TL_C,h_	Transmission loss of compact material of recycled rubber crumb at thickness *h*
TL_F,p,h_	Transmission loss of granular recycled rubber crumb at fraction *F*, pressure *p* and thickness *h*
*τ* *_f_*	Transmission coefficient at frequency *f* (unitless, –)
*τ*_avg_	Average transmission coefficient (unitless, –)
*TL* *_f_*	Transmission loss at frequency *f* (dB)
*TL*_avg_	Average transmission loss (dB)
*RTR_f_*	Relative transmission ratio at frequency *f* (unitless, –)

**Table 3:** Transmission coefficient for samples with a thickness of 2 cm at frequency *f* (in %).

**Freq (Hz)**	**F1**				**F2**				**Compact**
	**250 kPa**	**500 kPa**	**750 kPa**	**0 kPa**	**250 kPa**	**500 kPa**	**750 kPa**	**0 kPa**	
100	2.1	0.5	0.4	40.8	24.9	17.1	13.7	39.7	18.3
125	2.2	0.5	0.4	37.8	24.8	17.0	13.6	40.7	17.9
160	2.3	0.5	0.4	33.4	24.2	16.7	13.4	41.5	17.1
200	2.4	0.5	0.4	29.8	23.6	16.3	13.1	41.2	16.1
250	3.0	1.1	0.5	26.4	22.8	15.6	12.6	40.5	14.8
315	2.4	0.9	1.0	23.1	21.7	14.9	12.0	39.1	13.4
400	2.2	0.6	0.5	20.3	20.2	14.2	11.2	36.8	13.5
500	2.1	0.7	0.5	18.1	18.7	13.3	10.9	33.8	11.6
630	2.1	0.6	0.5	16.2	16.9	12.1	9.8	30.9	9.9
800	2.1	0.6	0.5	14.5	15.0	11.0	8.9	28.9	8.4
1000	2.0	0.6	0.5	13.5	13.6	10.2	8.3	25.7	7.4
1250	2.1	0.6	0.4	13.1	12.6	9.6	7.9	21.5	6.6
1600	2.2	0.5	0.3	13.2	12.1	9.4	7.9	17.7	6.1
2000	2.3	0.4	0.2	14.0	12.8	10.1	8.8	17.0	6.3
2500	2.2	0.3	0.2	15.0	14.4	11.5	10.0	19.6	7.0
*τ*_avg_ (in %)	2.25	0.60	0.43	21.96	18.57	13.27	10.81	31.64	11.62
*TL*_avg_ (in dB)	16.48	22.22	23.67	6.58	7.31	8.77	9.66	5.00	9.35
**Freq (Hz)**	**F3**				**F3**				**Compact**
	**250 kPa**	**500 kPa**	**750 kPa**	**0 kPa**	**250 kPa**	**500 kPa**	**750 kPa**	**0 kPa**	
100	45.9	43.3	33.7	58.2	57.5	52.0	49.2	64.8	18.3
125	45.0	42.6	33.1	56.9	56.6	51.1	48.3	64.3	17.9
160	43.4	41.0	31.8	54.3	54.7	49.3	46.3	62.3	17.1
200	41.5	39.2	30.2	52.0	52.4	47.0	43.9	60.1	16.1
250	38.6	37.7	28.1	51.5	49.1	44.0	40.9	57.2	14.8
315	36.4	34.8	25.7	47.4	45.3	40.5	37.2	53.8	13.4
400	33.3	31.8	22.9	43.0	41.5	36.3	33.3	49.2	13.5
500	29.4	28.9	21.1	39.7	37.0	32.6	29.6	44.4	11.6
630	25.8	25.6	18.2	35.4	32.3	28.3	25.6	39.3	9.9
800	21.9	22.2	15.5	30.4	27.5	24.0	21.8	33.6	8.4
1000	19.0	19.4	13.7	26.5	23.6	20.6	19.0	28.8	7.4
1250	16.7	17.4	12.5	23.4	20.5	17.9	16.9	24.8	6.6
1600	15.3	16.6	11.9	21.1	18.2	16.2	15.8	21.7	6.1
2000	15.9	17.6	12.8	21.1	18.2	16.9	17.1	21.5	6.3
2500	17.9	19.7	14.9	23.1	20.4	19.8	20.7	23.8	7.0
*τ*_avg_ (in %)	29.74	29.20	21.74	38.95	36.98	33.11	31.03	43.31	11.62
*TL*_avg_ (in dB)	5.27	5.35	6.63	4.09	4.32	4.80	5.08	3.63	9.35

**Table 4:** Transmission coefficient for samples with a thickness of 4.5 cm at frequency *f* (in %).

**Freq (Hz)**	**F1**				**F2**				**Compact**
	**250 kPa**	**500 kPa**	**750 kPa**	**0 kPa**	**250 kPa**	**500 kPa**	**750 kPa**	**0 kPa**	
100	0.23	0.06	0.07	12.93	6.83	4.53	3.20	22.63	7.98
125	0.21	0.05	0.07	11.46	6.77	4.54	3.19	23.65	7.91
160	0.21	0.05	0.07	9.93	6.63	4.45	3.14	24.07	7.41
200	0.29	0.05	0.14	8.79	6.65	4.62	3.08	23.53	6.87
250	0.31	0.23	0.16	7.82	6.90	4.74	3.32	22.67	6.70
315	0.33	0.10	0.08	7.03	6.18	4.23	3.23	21.35	6.97
400	0.29	0.08	0.08	6.39	5.80	4.07	2.96	19.60	5.82
500	0.19	0.06	0.06	6.01	5.56	3.94	2.92	17.88	5.09
630	0.15	0.05	0.04	5.70	5.34	3.84	2.86	16.80	4.72
800	0.09	0.03	0.02	5.46	5.29	3.85	2.90	16.48	4.52
1000	0.05	0.01	0.01	5.27	5.57	4.07	3.07	15.57	4.66
1250	0.02	0.005	0.001	4.90	6.149	4.41	3.29	15.22	5.14
1600	0.01	0.002	0.002	3.97	6.339	4.32	3.11	16.35	5.02
2000	0.002	0.001	0.003	2.89	5.093	3.39	2.39	15.53	3.90
2500	0.002	0.001	0.002	2.21	3.941	2.73	1.91	11.72	2.73
*τ*_avg_ (in %)	0.159	0.051	0.053	6.72	5.94	4.12	2.97	18.87	5.70
*TL*_avg_ (in dB)	28.00	32.89	32.77	11.73	12.27	13.86	15.27	7.24	12.44
**Freq (Hz)**	**F3**				**F4**				**Compact**
	**250 kPa**	**500 kPa**	**750 kPa**	**0 kPa**	**250 kPa**	**500 kPa**	**750 kPa**	**0 kPa**	
100	29.51	19.57	15.40	48.96	37.91	33.39	26.04	54.23	7.98
125	28.68	18.97	14.76	47.73	36.48	31.93	24.68	52.50	7.91
160	26.89	17.87	13.78	45.10	33.82	29.48	22.75	49.59	7.41
200	25.18	16.72	12.74	42.29	31.07	27.01	20.75	46.20	6.87
250	23.04	15.78	11.49	38.69	27.91	24.29	18.35	42.42	6.70
315	20.71	14.35	10.24	34.90	25.17	21.72	15.89	38.02	6.97
400	18.12	12.80	9.05	30.86	21.84	18.76	14.06	33.31	5.82
500	15.98	11.55	8.52	27.44	18.80	16.30	12.54	29.32	5.09
630	14.24	10.49	7.45	24.20	16.47	14.25	10.81	25.44	4.72
800	12.86	9.72	6.95	21.71	14.78	12.84	9.81	22.41	4.52
1000	12.72	9.72	7.09	20.66	14.32	12.58	9.82	21.12	4.66
1250	14.164	10.71	8.30	21.88	15.72	13.90	11.33	22.32	5.14
1600	18.323	12.96	10.78	27.54	21.25	18.84	15.75	29.17	5.02
2000	19.898	12.35	10.31	32.89	25.49	21.97	17.25	37.62	3.90
2500	15.241	9.65	7.89	27.57	20.05	17.01	13.01	32.28	2.73
*τ*_avg_ (in %)	19.70	13.35	10.32	32.83	24.07	20.98	16.19	35.73	5.70
*TL*_avg_ (in dB)	7.05	8.68	9.86	4.84	6.18	6.79	7.91	4.47	12.44

**Table 5:** Transmission coefficient for samples with a thickness of 7 cm at frequency *f* (in %).

**Freq (Hz)**	**F1**				**F2**				**Compact**
	**250 kPa**	**500 kPa**	**750 kPa**	**0 kPa**	**250 kPa**	**500 kPa**	**750 kPa**	**0 kPa**	
100	0.04	0.02	0.02	8.6	3.2	1.5	0.9	16.3	0.5
125	0.04	0.02	0.02	7.6	3.2	1.5	0.8	17.4	0.5
160	0.03	0.02	0.01	6.7	3.1	1.5	0.8	17.2	0.4
200	0.02	0.01	0.01	6.0	3.0	1.4	0.8	16.8	0.4
250	0.02	0.01	0.01	5.5	2.9	1.4	0.8	16.1	0.4
315	0.04	0.01	0.01	5.1	3.1	1.4	0.8	15.2	0.4
400	0.05	0.03	0.02	4.8	3.1	1.5	1.1	14.1	0.3
500	0.02	0.02	0.04	4.5	3.0	1.7	0.9	13.3	0.4
630	0.005	0.01	0.01	4.2	3.1	1.6	0.9	13.2	0.3
800	0.003	0.00	0.01	3.7	3.1	1.6	0.8	14.0	0.3
1000	0.005	0.01	0.005	2.9	2.9	1.3	0.7	14.2	0.3
1250	0.005	0.004	0.004	2.2	2.3	1.0	0.5	13.8	0.2
1600	0.003	0.002	0.002	1.4	1.7	0.8	0.4	10.3	0.1
2000	0.002	0.001	0.001	1.0	1.4	0.6	0.3	7.2	0.0
2500	0.001	0.001	0.001	0.7	1.2	0.5	0.2	6.8	0.1
*τ*_avg_ (in %)	0.018	0.011	0.012	4.33	2.69	1.28	0.71	13.74	0.31
*TL*_avg_ (in dB)	37.34	39.42	39.29	13.64	15.69	18.92	21.49	8.62	25.14
**Freq (Hz)**	**F3**				**F4**				**Compact**
	**250 kPa**	**500 kPa**	**750 kPa**	**0 kPa**	**250 kPa**	**500 kPa**	**750 kPa**	**0 kPa**	
100	15.5	10.4	6.1	43.1	23.8	19.4	12.6	45.2	0.5
125	14.7	9.9	5.8	41.3	22.3	18.3	11.7	43.2	0.5
160	13.5	9.2	5.3	38.1	20.1	16.4	10.5	39.5	0.4
200	12.3	8.4	4.9	35.1	17.9	14.7	9.4	36.5	0.4
250	10.9	7.5	4.5	31.6	15.6	12.7	8.2	32.4	0.4
315	9.9	6.9	4.1	28.2	13.6	11.2	7.2	28.3	0.4
400	9.1	6.6	3.8	25.0	12.3	10.2	6.3	24.7	0.3
500	8.6	6.1	4.0	22.9	11.0	9.2	6.2	22.1	0.4
630	8.3	6.1	4.0	21.2	10.4	8.8	6.1	20.2	0.3
800	8.9	6.7	4.4	21.3	11.2	9.6	6.9	20.3	0.3
1000	10.5	8.2	5.0	24.3	13.9	12.1	8.8	23.6	0.3
1250	11.5	8.8	4.6	30.2	18.1	15.0	9.9	31.9	0.2
1600	8.5	6.4	3.4	28.2	14.1	10.9	6.7	31.4	0.1
2000	6.6	5.3	2.9	18.8	9.6	7.7	5.4	20.2	0.0
2500	6.7	5.4	2.7	17.0	10.0	8.5	6.1	18.7	0.1
*τ*_avg_ (in %)	10.38	7.46	4.39	28.41	14.93	12.31	8.14	29.2	0.31
*TL*_avg_ (in dB)	9.84	11.27	13.58	5.46	8.26	9.10	10.89	5.335	25.14

**Table 6:** Regression models—transmission loss (α = 0.05).

Parameters	Coefficients	Standardized Coefficients Beta	*t*-Stat	*p*-Value
Granular recycled rubber crumb—*Fraction 1* (*p*-value < 2 × 10^−16^), R^2^ = 0.820				
Intercept	1.221	−5.96 × 10^−18^	0.716	0.0371
p	1.902 × 10^−2^	0.436	5.384	2 × 10^−7^
h	2.437 × 10^2^	0.408	7.271	<10^−10^
Freq	4.158 × 10^−3^	0.219	6.561	<10^−10^
p × h	2.333 × 10^−1^	0.300	3.237	1.1 × 10^−3^
Granular recycled rubber crumb—*Fraction 2* (*p*-value < 2 × 10^−16^), R^2^ = 0.955				
Intercept	2.755	3.9 × 10^−16^	7.822	<10^−16^
p	1.562 × 10^−3^	0.086	2.156	3.10^−2^
h	93.09	0.377	13.315	<10^−16^
Freq	1.408 × 10^−3^	0.189	11.412	<10^−13^
p × h	2.115 × 10^−1^	0.656	14.154	<10^−13^
Granular recycled rubber crumb—*Fraction 3* (*p*-value < 2 × 10^−16^), R^2^ = 0.838				
Intercept	2.482 × 10^−1^	3.9 × 10^−16^	0.883	0.379
p	6.643 × 10^−3^	0.579	18.954	<10^−16^
h	91.69	0.609	18.930	<10^−16^
Freq	1.417 × 10^−3^	0.324	9.514	<10^−16^
Granular recycled rubber crumb—*Fraction 4* (*p*-value < 2 × 10^−16^), R^2^ = 0.808				
Intercept	5.317 × 10^−1^	3.8 × 10^−16^	2.109	0.036
p	3.809 × 10^−3^	0.415	11.921	<10^−16^
h	76.3	0.618	17.748	<10^−16^
Freq	1.75 × 10^−3^	0.466	13.387	<10^−16^
*Compact Sample* (*p*-value < 2 × 10^−16^), R^2^ = 0.862				
Intercept	2.75 × 10^−2^	2.2 × 10^−16^	0.024	0.981
h	3.12	0.923	3.171	<10^−16^
Freq	2.43 × 10^−3^	0.191	15.251	3 × 10^−3^

## Data Availability

The original contributions presented in the study are included in the article. Further inquiries can be directed to the corresponding author.
